# Rare double-hit with two translocations involving *IGH* both, with *BCL2* and *BCL3*, in a monoclonal B-cell lymphoma/leukemia

**DOI:** 10.1186/s13039-015-0203-y

**Published:** 2015-12-30

**Authors:** Roman Alpatov, Billie Carstens, Kimberly Harding, Carolyn Jarrett, Sudabeh Balakhani, Jessica Lincoln, Peter Brzeskiewicz, Yu Guo, Alex Ohene-Mobley, Jamie LeRoux, Veronica McDaniel, Lynne Meltesen, Diane Minka, Mahendra Patel, Cyrus Manavi, Karen Swisshelm

**Affiliations:** Colorado Genetics Laboratory, Department of Pathology, University of Colorado School of Medicine, 3055 Roslyn Street, Suite 200, Denver, CO 80238 USA; Halifax Medical Specialists PA, Roanoke Rapids, NC USA; Eastern Carolina Pathology Associates, 1705 Tarboro Street SW, Wilson, NC 27893 USA

**Keywords:** B-cell lymphoma/leukemia, Double *IGH;BCL2* and *IGH;BCL3* translocation, *ERCC2* duplication

## Abstract

**Background:**

Chronic Lymphocytic Leukemia (CLL) is a lymphoproliferative disease characterized by multiple recurring clonal cytogenetic anomalies and is the most common leukemia in adults. Chromosomal abnormalities associated with CLL include trisomy 12 and *IGH;BCL3* rearrangement [t(14;19)(q32;q13)] that juxtaposes a proto-oncogenic gene *BCL3* and an immunoglobulin heavy chain, a translocation that may be associated with shorter survival. In addition to the *IGH;BCL3* rearrangement, other translocations involving 14q32 locus are involved in various lymphoproliferative pathologies pointing toward the significance of *IGH* locus in oncogenic progression. Significantly, in the majority of B-cell neoplasms that carry an *IGH;BCL3* rearrangement, it is a sole translocation involving an *IGH* locus.

**Case Presentation:**

We report a patient who, in addition to trisomy 12, carried a rare double-hit translocation characterized by the *IGH;BCL3* translocation and an additional clonal *IGH;BCL2* translocation involving *IGH* and another proto-oncogene *BCL2*, t(14;18)(q32;q21), commonly found in follicular lymphoma. Further single nucleotide polymorphism (SNP) array-based analysis detected a duplication of the 58.8 kb region at 19q13.32 adjacent to the *BCL3 *translocation junction on chromosome 19q13. Interestingly, the duplicated region contained *ERCC2* gene, which encodes a DNA excision repair protein involved in the cancer-prone syndrome, xeroderma pigmentosum.

**Conclusions:**

Taken together our findings indicate the existence of double-translocation driven oncogenic events involving both *IGH* loci and proto-oncogenes *BCL2 *and *BCL3*. Importantly, the *IGH;BCL3* translocation was characterized by the duplication of the genomic region adjacent to *BCL3*, containing a major DNA repair factor, *ERCC2*.

**Electronic supplementary material:**

The online version of this article (doi:10.1186/s13039-015-0203-y) contains supplementary material, which is available to authorized users.

## Background

Chronic lymphocytic leukemia (CLL) is a genetically heterogeneous neoplasm characterized by the progressive accumulation of B cells in bone marrow, lymph nodes and blood. The progression of the disease is highly variable, ranging from the indolent state to the highly aggressive leukemia marked by short survival times. Numerous chromosomal abnormalities have been shown to contribute to CLL, including but not limited to trisomy 12, loss of 11q22-q23 containing the *ATM* gene, loss of 13q14.3 and 6q, loss of 17p13 containing *TP53* gene, and others [1].

A specific translocation [t(14;19)(q32;q13)] which juxtaposes the immunoglobulin heavy chain locus (*IGH*) sequence (HUGO, 14q32.33) and the gene encoding an anti-apoptotic protein *BCL3* resulting in overexpression of BCL3 [2], is of a particular interest because it does not occur frequently, and it is usually associated with shorter survival [1]. In addition, this translocation as well as other translocations involving the *IGH* locus, although found in CLL, have been also described in poorly clinicopathologically described B cell lymphomas, categorized as atypical CLL [3]. Another translocation involving *IGH* locus and an anti-apoptotic protein *BCL2*, t(14;18)(q32;q21), is considered a hallmark of aggressive lymphomas, such as follicular lymphomas (FL) [4, 5] and diffuse large B-cell lymphomas (DLBCL) [6–8].

We report a patient who was evaluated for leukocytosis with lymphocytosis, and who was found to have a marked bone marrow involvement by neoplastic lymphocytes showing a B-cell line of differentiation as determined by flow cytometry and immunohistochemistry. Morphologically, the lymphocytes were small to medium in size, and a subset showed knobby cytoplasmic blebbing. Further immunophenotypic studies ruled out involvement by typical CLL or mantle cell lymphoma in this patient, highlighting an atypical characteristic of this malignancy. Surprisingly, in addition to trisomy 12, which is commonly found in CLL, chromosome analysis at the haploid band resolution 300-400 revealed the presence of clones carrying a single t(14;19)(q32;q13) *IGH;BCL3* translocation or both, t(14;19)(q32;q13) *IGH;BCL3* and t(14;18)(q32;q21) *IGH;BCL2* translocations. Whereas single translocation events involving *IGH* and *BCL3* or *BCL2* loci can be detected in lymphoid cancers, double-hit translocations involving both *IGH;BCL2* and *IGH;BCL3* rearrangements in the same patient are exceptionally rare. Additionally, using SNP array analysis we detected a local microduplication event at the *BCL3* translocation junction on chromosome 19 involving a DNA repair factor *ERCC2*. An additional file describing experimental procedures is available (see Additional file 1). However, due to the nature of the SNP array platform we cannot exclude the possibility that *ERCC2* duplication occurred on the non-rearranged chromosome 19. Given the absence of classical CLL and mantle cell lymphoma immunophenotype, size and morphology of the neoplastic lymphocytes, and the presence of B-cell lymphoproliferative genetic markers characteristic of aggressive B-cell lymphomas, we favor the diagnosis of an aggressive monoclonal B-cell lymphoma/leukemia, likely B-prolymphocytic leukemia in this patient driven by the *IGH;BCL3* and *IGH;BCL2* mediated mechanisms.

## Case presentation

Our patient was an 82-year-old African-American female who presented to her oncologist for leukocytosis. Complete blood count (CBC) data showed a white blood cell (WBC) count of 24.5 K/ MicroL with relative and absolute lymphocytosis of 70 % and 17.2K/ MicroL respectively. A bone marrow biopsy and aspirate was performed and the specimen was sent to the hematopathologist for evaluation. Flow cytometric analysis showed 40 % monoclonal B-cells with lambda light chain restriction of moderate intensity with dim CD5 co-expression and the following immunophenotype: CD10-, CD19+, CD20+, CD200-, CD23 +/- (dim), FMC7 +/- (dim), CD38-, CD25-, CD103- and CD11c-.

**Fig. 1:**
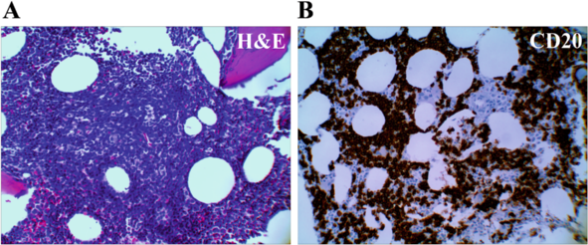
**a** H&E staining of the bone marrow showing marked interstitial involvement by medium sized cells with small but conspicuous nucleoli. **b** CD20 immunohistochemistry highlights marked involvement of the bone marrow by abnormal B-cells

By histology the bone marrow showed marked involvement by lymphocytes with interstitial pattern of distribution estimated at 80–90 % of the total marrow cellularity. H&E staining, CD20 and Pax5 immunohistochemistry confirmed the nature of the lymphocytes consistent with B-cell line of differentiation (Fig. 1a and b and data not shown). No lymphoid aggregate formation was seen. Morphologically the lymphocytes were small to medium in size and showed clumped nuclear chromatin with small but conspicuous nucleoli and no morphologic features suggestive of involvement by DLBCL, Burkitt lymphoma or other high-grade aggressive B-cell lymphomas.

By immunohistochemistry the B-cells were negative for Cyclin-D1 and, additionally, they were negative for Sox-11, arguing against the diagnosis of mantle cell lymphoma or Cyclin-D1 negative mantle cell lymphoma, respectively. Evaluation of the aspirate smear showed the lymphocytes with occasional small knobby cytoplasmic blebbing but no discernible villous hairy projections.

Overall, based on the immunophenotypic finding by flow cytometric analysis, it is unlikely that this lymphoma represents typical CLL, since it not only expresses monoclonal light chain with moderate intensity, it also shows weak CD23 expression and is negative for CD200. The possibility of mantle cell lymphoma and Cyclin D-1 negative mantle cell lymphoma was considered and further evaluated but ruled out by negative staining with Cyclin D1 and Sox-11 immunohistochemistry respectively. Given the morphologic observation of knobby cytoplasmic blebbing, the diagnosis of B-PLL (B- prolymphocytic leukemia) was considered a strong possibility.

**Fig. 2:**
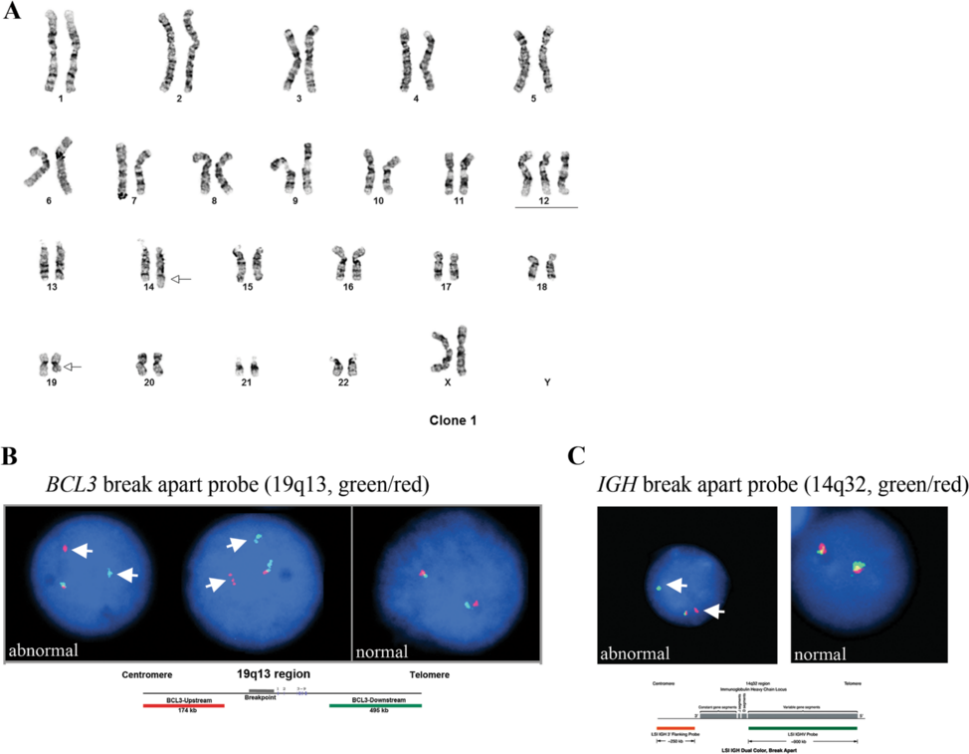
**a** Karyotype of the first clone. The first clone (stemline [sl]), six cells, contained a translocation between the long (q) arms of chromosomes 14 and 19 (arrows), and gain of one copy of chromosome 12 (underlined). **b** *BCL3* break-apart FISH probe (green/red) indicates rearrangement of a *BCL3* locus in the first clone (arrows). Normal FISH signal is shown on the right side of the panel for comparison. **c** *IGH* break-apart FISH probe (green/red) indicates rearrangement of an *IGH* locus in the first clone (arrows). Normal FISH signal is shown on the right side of the panel for comparison

**Fig. 3:**
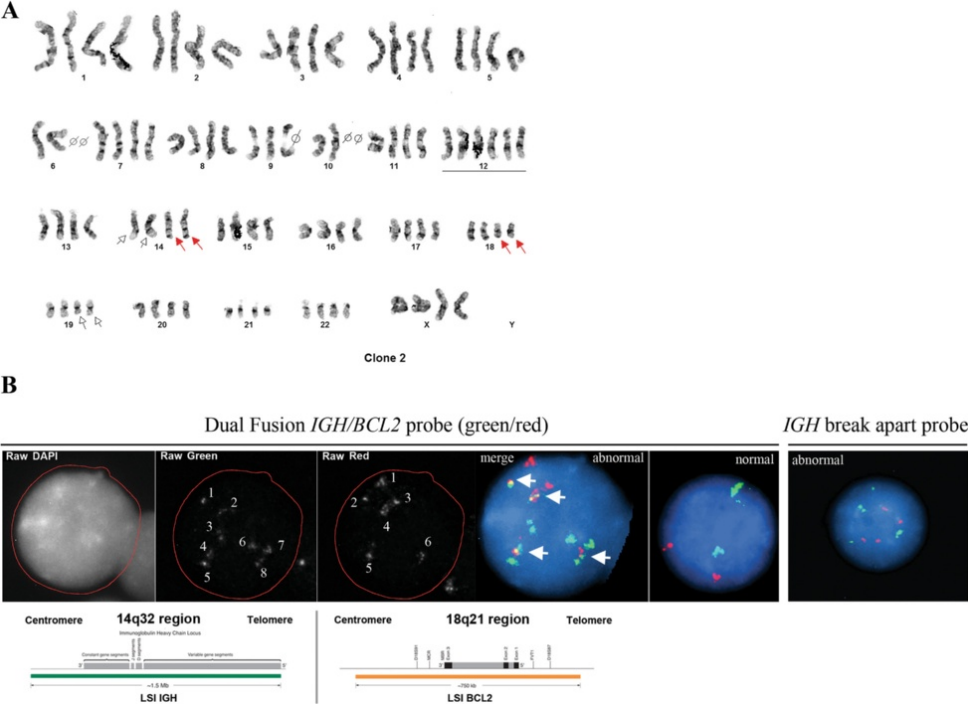
**a** Representative karyotype of the second clone. The second clone, a composite of two cells, was a doubling of the stemline clone (the first clone) with two copies of a translocation between 14q and 18q (*IGH;BCL2*, red arrows), in addition to a 14q and 19q translocation (*IGH;BCL3*, white arrows). Six copies of chromosome 12 are underlined. **b** FISH analysis of the second clone using dual fusion *IGH;BCL2* probe (green/red). Black and white image for *IGH* signal shows 8 loci indicating that all *IGH* sequences are rearranged and present on derivative chromosomes 14, 18, and 19. Black and white image for *BCL2* signal shows 6 loci which represent two intact and two derivative chromosomes 18, and two derivative chromosomes 14. Merged image shows green *IGH* signals and red *BCL2* signals which overlap in four loci, arrows (two copies of derivative chromosome 14 and two copies of derivative chromosome 18). Right panel, *IGH* break-apart probe (green/red) signal showing four green and four red foci indicative of the presence of four rearranged *IGH* loci

**Fig. 4:**
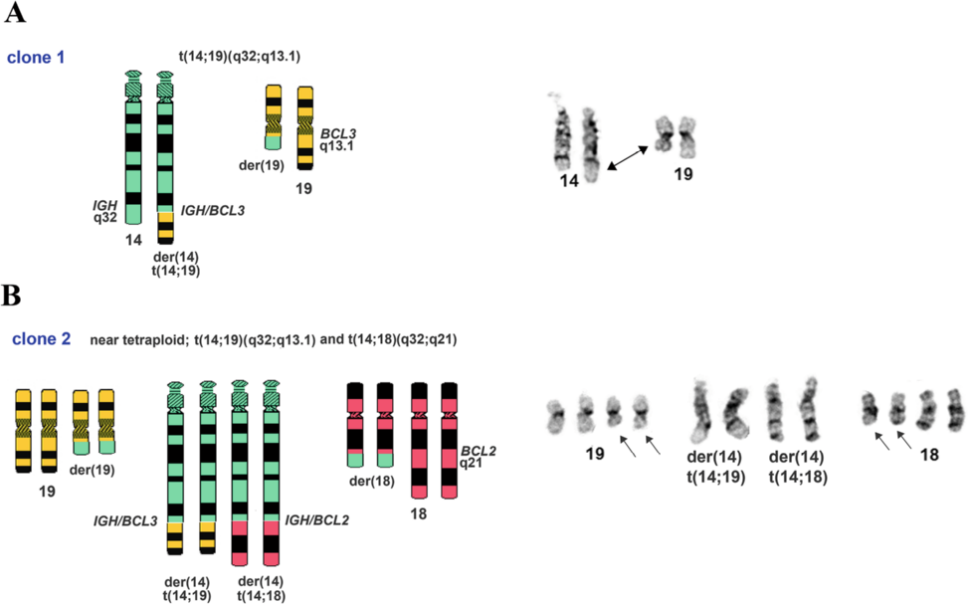
Schematic representation of the rearrangements described in clone 1 and clone 2. Chromosomes involved in rearrangements are shown on the right. **a** Rearrangements in clone 1 involving *IGH* (14q32) and *BCL3* (19q13.1) which results in two derivative chromosomes (der(19) and der(14). Rearranged chromosomes 14 and 19 are indicated by an arrow on the right. **b** Rearrangements in clone 2 which includes chromosome duplication event and *IGH* (14q32) and *BCL2* (18q21) translocation in addition to *IGH;BCL3* rearrangements. Therefore clone 2 contains 8 derivative chromosomes (a pair of each der(19), der(18), der(14)t(14;19), and der(14)t(14;18)). Rearranged chromosomes 18 and 19 are indicated by the arrows on the right

Cytogenetic studies of bone marrow preparations from this patient revealed two related abnormal clones in eight of twenty-two metaphase spreads examined. The first clone (stemline [sl]), six cells, contained a translocation between the long (q) arm of chromosomes 14 and 19 (Fig. 2a, arrows), and gain of one copy of chromosome 12 (underlined). The second clone, a composite of two cells, was a doubling of the stemline clone (the first clone) with two copies of an additional translocation between 14q and 18q (Fig. 3a, red arrows, six copies of chromosome 12 are underlined). The remaining fourteen cells contained a normal karyotype. Additionally, FISH studies using *BCL3* and *IGH* break-apart probes confirmed *BCL3* and *IGH* translocations in the first clone (Fig. 2b and c, arrows). With respect to the second clone, *IGH;BCL2* dual-fusion probe picked up eight signals for *IGH* (Fig. 3b, black and white *IGH* image) which, in combination with karyotype data, was indicative of the presence of eight *IGH* derivative translocation products. This suggests that all *IGH* loci are rearranged in the second clone in addition to the near duplication of the chromosome content. *BCL2* probe picked up six *BCL2* signals (Fig. 3b, black and white *BCL2* image) indicating on the presence of four translocated *BCL2* loci and two intact *BCL2* loci from the non-translocated chromosome 18. Importantly, *IGH* and *BCL2* signals formed four fusions (Fig. 3b, “merge” panel, arrows) confirming two derivative *IGH;BCL2* loci on chromosomes 14 and chromosome 18. In addition, *IGH* break-apart probe confirmed the presence of four translocated *IGH* loci (Fig. 3b, right panel, four red and four green signals). In summary, first clone was characterized by the *IGH;BCL3* translocation event resulting in derivative chromosomes, 14 and 19 (Fig. 4a) and second clone was characterized by the presence of *IGH;BCL2* translocation in addition to *IGH;BCL3* translocation and near tetraploid chromosome content resulting in four derivative chromosomes 14 and two derivative chromosomes 18 and 19 (Fig. 4b).

The patient’s ISCN was 47,XX,+12,t(14;19)(q32;q13.1)[6]/89 ~ 90,slx2,t(14;18)(q32;q21)*x*2[cp2]/46,XX[14].nuc ish(D11Z1,ATM)*x*2[198],(CCND1x2,IGHx3)[78/200],(D12Z3x3,D13S319x2)[117/200],(IGHx2)(5'IGH sep 3'IGHx1)[153/300]/(IGHx4)(5'IGH sep 3'IGHx4)[1/300],(IGHx3,BCL2x2)[123/300]/(IGHx8,BCL2x6)(IGH con BCL2x4)[1/300],(TP53,D17Z1)*x*2[189],(BCL3x2)(5'BCL3 sep 3'BCL3x1)[99/200].

**Fig. 5:**
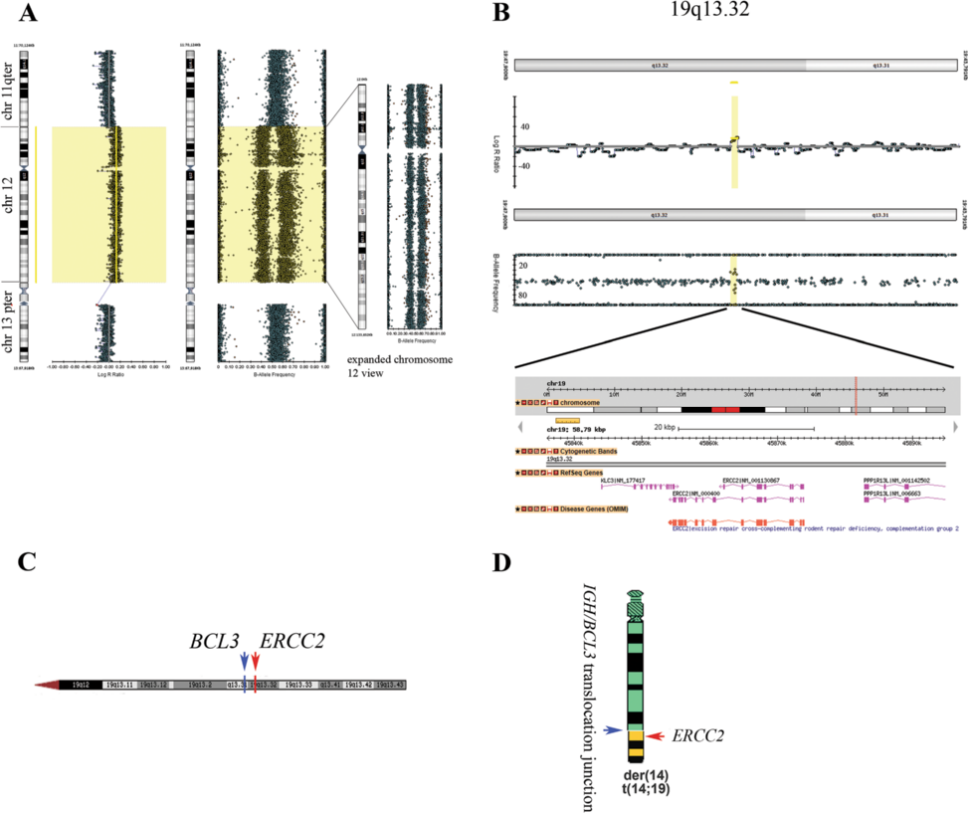
Single Nucleotide Polymorphism (SNP) array analysis. **a** Confirmation of an extra copy of chromosome 12. In the Log R ratio panel (left) chromosomal boundaries between the q terminal of chromosome 11, chromosome 12, and the p terminal of chromosome 13 are demarcated by lines. The expanded chromosome 12 view (right), shows an isolated view of chromosome 12 (B-Allele frequency). **b** Microduplication of the genomic region containing *ERCC2* gene. **c** Genomic mapping of *ERCC2* gene located in the 19q13.32 cytogenetic band, proximal to the *BCL3* gene locus (19q13.31). **d** Placement of the *ERCC2* gene relative to the hypothesized *IGH;BCL3* rearrangement junction depicted in the schematics (the possibility exists that *ERCC2* duplication occurred on the non-translocated chromosome 19 as stated in text)

Further studies using single nucleotide polymorphism arrays on the patient’s DNA sample detected the presence of an extra chromosome 12, confirming our karyotype analysis (Fig. 5a). Interestingly, we also detected a microduplication event at the cytogenetically defined *BCL3* translocation junction (19q13.1) containing a major nucleotide excision repair gene *ERCC2* (ISCN arr[hg19](12)X3,19q13.32(45,835,983-45,894,768)x3), (Fig. 5b-d). Polymorphisms in this gene are associated with xeroderma pigmentosum (XP), XP associated with Cockayne syndrome (XP/CS), and trichothiodystrophy (TTD) [9]. However, we do not exclude the possibility that *ERCC2* duplication occurred on the intact chromosome 19.

## Conclusions

In this report we describe a case of a monoclonal B-cell lymphoma/leukemia with extensive bone marrow involvement and CLL-like immunophenotype showing CD5 expression by flow cytometric analysis and furthermore displaying a double translocation event between *IGH* loci and both *BCL3* and *BCL2* gene loci. Additionally, we detected a microduplication event in the genomic locus adjacent to *BCL3* gene involving a nucleotide excision repair protein *ERCC2*. Our karyotype analysis identified two abnormal clones in this patient. The first clone contained, in addition to trisomy 12, a single cytogenetically defined translocation t(14;19)(q32;q13) involving *IGH;BCL3* loci. The second clone represented a near duplication of the chromosome content of the first clone and the presence of two copies of an additional translocation involving *IGH* locus and *BCL2* gene t(14;18)(q32;q21). Therefore, all *IGH* loci located on the chromosome 14 were rearranged in the second clone which was confirmed by our FISH studies.

These results are consistent with a neoplastic process in this patient’s bone marrow specimen. Importantly, the translocation t(14;19)(q32;q13) involving *IGH* and *BCL3* loci, which results in altered *BCL3* expression, has been described in aggressive forms of lymphomas and atypical CLLs [3, 10]. The translocation t(14;18)(q32;q21) is a recurring abnormality in B-cell lymphomas. This translocation places the oncogene *BCL2* on 18q21 within the immunoglobulin heavy chain locus on 14q32 and therefore deregulates the *BCL2* function. Juxtaposition of both *BCL3* and *BCL2* genes next to the active *IGH* locus leads to their altered expression, decreased apoptosis, and leukemia progression [10, 11]. Concurrent rearrangements of *BCL2*(18q21) and *BCL3*(19q13) have been reported in atypical chronic lymphocytic leukemia at Richter's transformation [12]. Both *BCL3* and *BCL2* are proto-oncogenes, however mechanistically, BCL2 functions as a key regulator of apoptosis through the mitochondrial pathways [11], whereas BCL3 is a predominantly nuclear protein recruited to the NFκB-responsive promoters where it regulates apoptotic program [13], suggesting that rearrangements of BCL2 and BCL3 may differentially modulate leukemic progression through distinct molecular pathways. The appearance of the *IGH;BCL3* clone followed by the genome near duplication event and the appearance of the second clone containing both *IGH;BCL3* and *IGH;BCL2* rearrangements in our patient might, therefore, have an additive effect on the development of this neoplasm.

*ERCC2* duplication in this patient is an intriguing finding, because of the role of *ERCC2* in DNA nucleotide excision repair process. Specific *ERCC2* polymorphisms are implicated in susceptibility to melanoma [14] as well as triple negative breast cancer [15]. However, according to the Database of Genomic Variants (http://dgv.tcag.ca/dgv/app/home), duplications of *ERCC2* genomic region are also found in healthy individuals. Therefore, the significance of *ERCC2* duplication in context of *IGH;BCL3* and *IGH;BCL2* rearrangements requires further investigation. Additionally, the existence of the microduplication event at the major translocation junction supports the previously reported genomic instability events at the translocation breakpoints and emphasizes the significance of the molecular analysis of sequences adjacent to the cytogenetically defined translocation junctions including balanced rearrangements [16].

## Consent

Written informed consent was obtained from the patient for publication of this case report and any accompanying images. A copy of the written consent is available for review by the Editor-in-Chief of this journal.

## Additional file

Additional file 1:
**Experimental procedures.** (PDF 269 kb)
