# Supplementary material for: Rare double-hit with two translocations involving IGH both, with BCL2 and BCL3, in a monoclonal B-cell lymphoma/leukemia
Source: Mol Cytogenet. 2015 Dec 30;8:101. doi: 10.1186/s13039-015-0203-y (PMC4696310; doi:10.1186/s13039-015-0203-y)
Supplement: Additional file 1: — Experimental procedures. (PDF 269 kb) [file 13039_2015_203_MOESM1_ESM.pdf]

Experimental Procedures.

Flow Cytometry.

The bone marrow aspirate was processed and analyzed following the laboratory standard operating procedures. The flow cytometric analysis was done with a 3-tube 10-color panel (see Table) on a Gallios flow cytometer from Beckman Coulter. The panel is routinely used for lymphoma diagnosis.

	FITC	PE	ECD	PC5.5	PC7	APC	APC-AF700	APC-AF750	Pacific Blue	Krome-Orange
Tube 1	pKappa	pLambda	CD10	CD38	CD34	CD20	CD23	CD19	CD5	CD45
Tube 2	CD8	CD2	CD7	CD3	CD34	CD56	CD16	CD4	CD5	CD45
Tube 3	CD103	CD200	CD5	CD3	CD11c	CD22	CD25	CD19	FMC-7	CD45

CD103, CD200 antibodies are from BD Biosciences, all the other antibodies are from Beckman Coulter.

Cytogenetic Studies.

Cytogenetic studies were performed on overnight unstimulated, three day unstimulated and two day CpG-oligonucleotide DSP 30 plus interleukin-2 stimulated bone marrow preparations. Twenty two metaphase spreads were analyzed at the haploid band resolution 300-400.

Fluorescence In Situ Hybridization (FISH).

A dual color assay using the break-apart translocation probe *BCL3* was performed to look for a rearrangement of the *BCL3* locus at 19q13 using 5'BCL3(19q13)/3'BCL3(19q13) FISH probe (Abbott), 200 interphase cells were analyzed. A dual color assay using the break-apart *IGH* translocation FISH probe 5'IGH(14q32.3)/3'IGH(14q32.3) (Abbott) was used to detect *IGH* translocation, 300 interphase cells

were analyzed. A dual color, dual fusion assay was performed to look for the *IGH;BCL2* rearrangement associated with the (14;18)(q32.3;q21) translocation using IGH(14q32)/ BCL2(18q21) FISH probe (Abbott), 300 interphase cells were analyzed. This assay detects the fusion products of both translocation partners.

Single Nucleotide Polymorphism Array.

Chromosomal microarray (CMA) was performed on DNA extracted from the same bone marrow sample used for karyotyping and FISH assays and processed on the Illumina® CytoSNP-850K bead array according to the manufacturer instructions. The CytoSNP-850K array targets more than 3,262 disease regions. Data analysis is based on the Genome Reference Consortium Human genome Build 37 [hg19] produced by the National Center for Biotechnology Information (NCBI). Copy number variant regions are analyzed within the Database of Genomic Variants, http://projects.tcag.ca/variation/.
